# Integrating electrical impedance tomography and transpulmonary pressure monitoring to personalize PEEP in hypoxemic patients undergoing pressure support ventilation

**DOI:** 10.1186/s13054-022-04198-4

**Published:** 2022-10-18

**Authors:** Douglas Slobod, Marco Leali, Elena Spinelli, Domenico Luca Grieco, Savino Spadaro, Tommaso Mauri

**Affiliations:** 1 Department of Anesthesia, Critical Care and Emergency, Fondazione Istituto Di Ricovero E Cura a Carattere Scientifico Ca’ Granda, Ospedale Maggiore Policlinico, Via F. Sforza 35, 20122 Milan, Italy; 2 Department of Critical Care Medicine, McGill University, Montreal, QC Canada; 3 Department of Pathophysiology and Transplantation, University of Milan, Milan, Italy; 4 Department of Emergency, Intensive Care Medicine and Anesthesia, Fondazione Policlinico Universitario A. Gemelli IRCCS, Rome, Italy; 5 Anesthesia and Intensive Care Unit, Department of Translational Medicine, University of Ferrara, Ferrara, Italy

**Keywords:** Pressure support ventilation, Electrical impedance tomography, Personalized positive end-expiratory pressure

## Abstract

Monitoring with electrical impedance tomography (EIT) during a decremental PEEP trial has been used to identify the PEEP that yields the optimal balance of pulmonary overdistension and collapse. This method is based on pixel-level changes in respiratory system compliance and depends on fixed or measured airway driving pressure. We developed a novel approach to quantify overdistension and collapse during pressure support ventilation (PSV) by integrating transpulmonary pressure and EIT monitoring and performed pilot tests in three hypoxemic patients. We report that our experimental approach is feasible and capable of identifying a PEEP that balances overdistension and collapse in intubated hypoxemic patients undergoing PSV.

## Introduction

Selection of physiology-based personalized positive end-expiratory pressure (PEEP) is key to the management of intubated patients with acute hypoxemic respiratory failure. PEEP should balance alveolar recruitment (which decreases volu- and atelectrauma) with the risk of overdistension (which increases barotrauma) [1].

Conventional pulmonary monitoring with electrical impedance tomography (EIT) during a decremental PEEP trial has been used to quantify regional overdistension and collapse at each level. The PEEP that yields the lowest difference (crossover PEEP) between the two phenomena could be considered as an optimal balance. This method is based on pixel-level changes in respiratory system compliance and depends on fixed or measured driving pressure during the decremental trial [2, 3]. Considering only the airway pressure (Paw) during assisted ventilation can be flawed because the driving pressure is composed of both ventilator support and the patient’s inspiratory effort.

We developed a modified approach to quantify lung overdistension and collapse by integrating esophageal pressure (Pes) and EIT monitoring during a decremental PEEP trial. We assessed pixel-level changes in lung compliance by using the dynamic transpulmonary driving pressure (∆P_Ldyn_) instead of airway driving pressure. Here, we describe the novel methodology and report the feasibility of the modified approach based on pilot tests in three intubated patients with acute hypoxemic respiratory failure of different etiologies undergoing pressure support ventilation (PSV).

## Methods

### Novel experimental approach to identify crossover PEEP during PSV

Paw, measured at the endotracheal tube, and Pes were recorded simultaneously with the EIT signal. A transpulmonary pressure (P_L_) waveform was generated offline as the difference between Paw and Pes. Automated breath detection was performed on P_L_ tracings by adapting a previously published algorithm [4]. Within each breath, ∆P_Ldyn_ was calculated as the difference between the maximal P_L_ (peak inspiration) and P_L_ at end-expiration.

The EIT signal was expressed as a relative impedance change from end-expiration (∆Z). Linear regression between impedance within each pixel and global impedance in the whole image was estimated. Pixels with a regression coefficient of at least 20% of the maximum at any step were classified as ventilated [5]. Within each ventilated pixel, the tidal variation in impedance (∆Ztidal,px) was calculated as the difference between the maximum and minimum value of the impedance signal for each breath.

Dynamic lung compliance for each breath and for each pixel (C_L_,px) was calculated as:

1 $${\text{C}}_{{\text{L}}} ,\;{\text{px}} = \Delta {\text{Z}}\;{\text{tidal}},{\text{px}}/\Delta {\text{P}}_{{{\text{Ldyn}}}}$$

To ensure that the inadvertent inclusion of brief artifacts (e.g., patient–ventilator dyssynchronies, air leaks, coughing) leading to incorrect breath detection or compliance estimations would not affect our findings, breaths yielding a global dynamic lung compliance value above or below 3 scaled mean absolute deviations from the median were rejected as outliers. Then, for each PEEP step, segments comprising ~ 10 breaths were manually selected toward the end of the step and compliance for each pixel was averaged over breaths to obtain a compliance map. As previously described [2], relative compliance for each pixel and step was calculated as the percentage departure from the highest compliance obtained at any step within that pixel. Each relative compliance change was categorized as lung collapse or overdistension according to the decreasing PEEP trend [2, 3]. The crossover PEEP was defined as the tested PEEP step that yielded the smallest difference between the percentages of overdistension and collapse in the whole image. MATLAB R2021a was used to implement the algorithm.

### Patients test

Data were recorded from three intubated hypoxemic patients already monitored by EIT and esophageal pressure and undergoing a decremental PEEP trial during PSV for clinical purposes. The trial was performed by setting PEEP to 12 cm H_2_O and decreasing it to 6 cm H_2_O, in 2 cm H_2_O steps, maintained for 2 min each. The PEEP range was selected considering the severity of the respiratory failure and the assisted ventilation mode. EIT data were continuously recorded at a 50 Hz sampling frequency via a 16-electrode belt placed around the chest in the axial plane at the 5th intercostal space (Dräger, Lübeck, Germany). At the end of the trial, data were downloaded and analyzed offline using the Dräger EIT Data Analysis Tool version 6.3 (Dräger, Lübeck, Germany).

## Results

Patient 1 was a 43-year-old woman with primary graft dysfunction following a bilateral lung transplant; her PaO_2_/FiO_2_ was 240 mmHg on clinical PSV 8 cm H_2_O and PEEP 8 cm H_2_O. Patient 2 was a 46-year-old man with extensive left lung pneumonia and empyema; his PaO_2_/FiO_2_ was 188 mmHg with PSV 6 cm H_2_O and PEEP 10 cm H_2_O. Patient 3 was a 72-year-old woman with ARDS due to pneumonia; her PaO_2_/FiO_2_ was 204 mmHg with PSV 2 cm H_2_O and PEEP 12 cm H_2_O.

Respiratory parameters and the results from the experimental combined EIT-∆P_Ldyn_ analyses during the decremental trial are displayed in Table 1.

**Table 1:** Respiratory mechanics and EIT data obtained during the decremental PEEP trial

	PEEP			
	12 cm H_2_O	10 cm H_2_O	8 cm H_2_O	6 cm H_2_O
*Respiratory mechanics*				
∆Pes (cmH_2_O)				
Patient 1	2.7	2.1	1.4	5.5
Patient 2	4.4	4.9	4.5	3.5
Patient 3	3.2	3.2	3.2	3.1
Dynamic ∆P_L_ (cmH_2_O)				
Patient 1	10.7	10.3	9.6	13.6
Patient 2	10.9	11.4	11.0	9.9
Patient 3	5.8	5.6	5.5	5.6
Dynamic lung compliance (ml/cmH_2_O)				
Patient 1	30	31	34	23
Patient 2	32	35	34	34
Patient 3	59	64	65	59
*EIT data*				
Overdistension experimental method (%)				
Patient 1	18	10	3	0
Patient 2	8	5	3	0
Patient 3	12	5	0	0
Collapse experimental method (%)				
Patient 1	0	1	2	28
Patient 2	0	0	0	1
Patient 3	0	0	1	10
Crossover PEEP experimental method				
Patient 1			8	
Patient 2				6
Patient 3			8	

*EIT* electrical impedance tomography, *PEEP* positive end-expiratory pressure, ∆Pes = change in esophageal pressure, ∆*P*_*L*_ = dynamic transpulmonary driving pressure

**Fig. 1:**
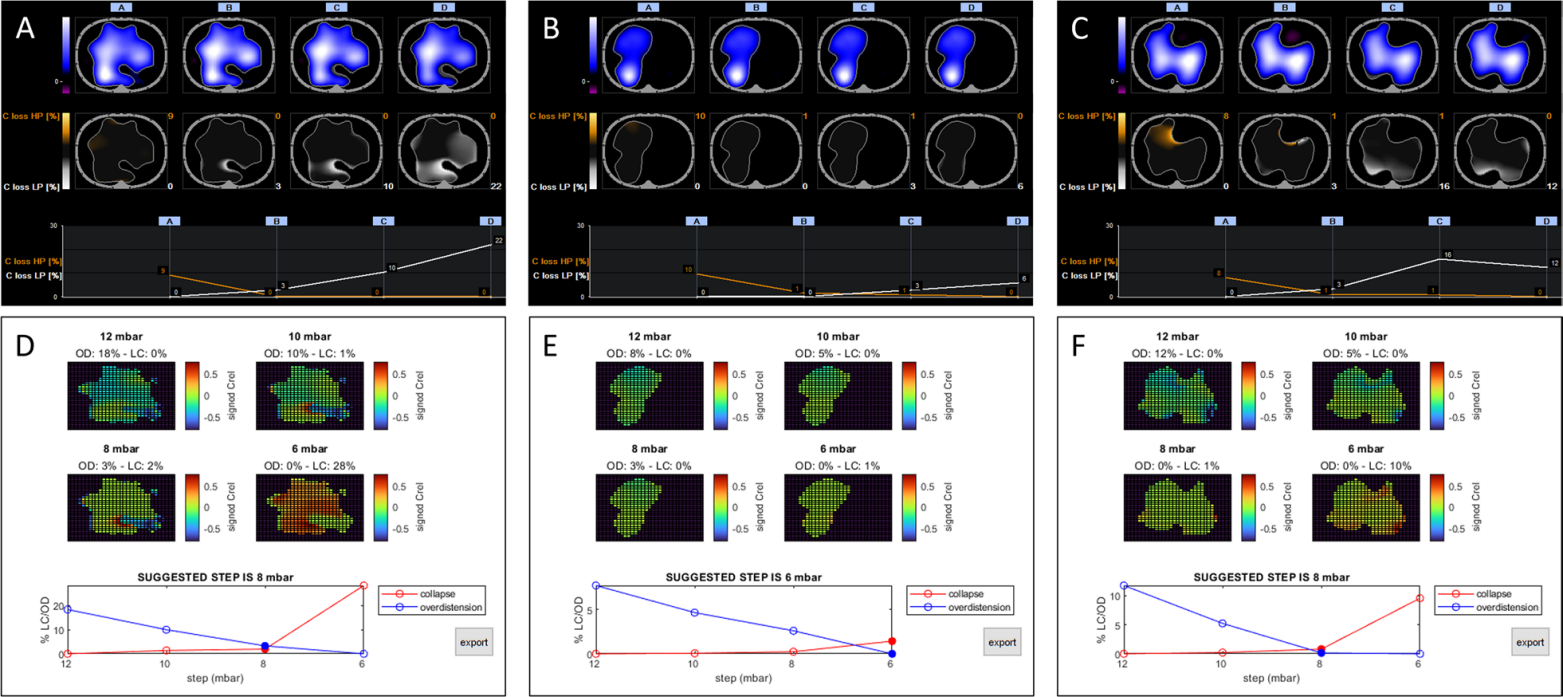
Decremental PEEP trial performed with the conventional and experimental approach. Results from a decremental PEEP trial performed during pressure support ventilation in patients 1, 2, and 3 using the conventional approach that assumes a fixed airway driving pressure (Panels A, B, and C) and the experimental approach that uses the measured dynamic transpulmonary driving pressure (Panels D, E, and F). Regional compliance maps and the percentage of overdistension and collapse are plotted against PEEP steps for both approaches. *PEEP* Positive end-expiratory pressure

Inspiratory effort (and thus ∆*P*_Ldyn_) varied across the PEEP trial, confirming the physiological rationale behind our novel method. Identification of the PEEP level associated with the lowest difference between overdistension and collapse was feasible in all the three patients (Fig. 1). Explorative data also showed that, in all patients, the PEEP that balanced overdistension and collapse based on the experimental approach was lower than the PEEP identified using the conventional approach (Fig. 1).

## Discussion

We developed a novel experimental approach to select personalized PEEP by integrating EIT and Pes monitoring in patients undergoing PSV. Our approach could be different from the conventional approach because it uses measured ∆P_Ldyn_ instead of airway driving pressure to calculate the extent of overdistension and collapse at each PEEP. Pilot testing in three patients with different severities and etiologies of hypoxemic respiratory failure demonstrates that the novel method is physiologically rational and clinically feasible.

Our experimental approach may represent a more accurate assessment of regional lung mechanics in the setting of assisted ventilation as it accounts for variations in the patient effort that can occur due to changes in PEEP [6] and uses ∆P_Ldyn_ instead of the airway driving pressure. Indeed, the experimental crossover PEEP tended to be associated with the lowest inspiratory effort and, thus, ∆P_Ldyn_. This suggests that the PEEP which results in the lowest difference between lung collapse and overdistension may minimize respiratory drive and effort for a given inspiratory support level [7].

Our method can be applied at the bedside in patients in whom Pes is monitored. However, it also has some limitations: It uses a dynamic measurement of ∆P_L_, and should be used with caution when airway resistance might vary significantly between PEEP steps (i.e., very high PEEP or obstructive lung disease); peristalsis waves should be carefully monitored during the decremental PEEP trial and, theoretically, each step might need to be maintained longer than during passive conditions; it is not feasible in patients with contraindication to Pes monitoring (e.g., esophageal surgery or bleeding). Finally, our pilot tests studied a relatively narrow range of PEEP steps and we acknowledge the possibility that higher lung compliance may have existed at a PEEP > 12 cm H_2_O.

To conclude, in patients undergoing PSV, a simultaneous EIT- and Pes-based decremental PEEP titration method that accounts for variations in the inspiratory effort is feasible.

## Data Availability

The datasets used and analyzed in the current report are available from the corresponding author on reasonable request.

## Abbreviations

PEEP: Positive end-expiratory pressure
EIT: Electrical impedance tomography
Paw: Airway pressure
Pes: Esophageal pressure
∆P_Ldyn_: Dynamic transpulmonary driving pressure
PSV: Pressure support ventilation
P_L_: Transpulmonary pressure
∆Z: Impedance change
∆Ztidal,px: Tidal impedance change per pixel
C_L_,px: Dynamic lung compliance per pixel
